# The Role of Malnutrition, Nutritional Deficiencies and Eating Disorders in Patients with Takotsubo Syndrome—Scoping Review

**DOI:** 10.3390/nu18142284

**Published:** 2026-07-12

**Authors:** Dominika Wiśniewska, Martyna Winiarska, Sabina Krupa-Nurcek

**Affiliations:** 1 Faculty of Medicine, Collegium Medicum, University of Rzeszów, 35-959 Rzeszow, Poland; dominika.wisniewska.001@gmail.com (D.W.); martynawiniarska44@gmail.com (M.W.); 2 Department of Surgery, Faculty of Medicine, Collegium Medicum, University of Rzeszów, 35-310 Rzeszow, Poland

**Keywords:** takotsubo syndrome, malnutrition, nutritional deficiencies, eating disorders, renutrition syndrome, cardiac complications

## Abstract

Objectives: Takotsubo syndrome (TTS) is an acute, often reversible disorder of left ventricular systolic function, the pathophysiology of which is complex and still being studied. A growing body of clinical research indicates that patients’ nutritional status may substantially influence the development and trajectory of Takotsubo syndrome. This review aims to systematically identify, map, and synthesize the existing scientific evidence on how malnutrition, nutrient deficiencies, and eating disorders contribute to Takotsubo syndrome (TTS), with particular attention being paid to the underlying pathophysiological mechanisms, predisposing factors, clinical manifestations, and prognostic implications. Methods: The scoping review was carried out following the methodological framework of the Joanna Briggs Institute (JBI) and in alignment with the PRISMA-ScR reporting standards. A comprehensive search for descriptors and keywords was performed across six electronic databases: PubMed, Scopus, Web of Science, EBSCO (MEDLINE Complete), Cochrane Library, and Google Scholar on 23–30 April 2026. Systematic reviews and meta-analyses were screened only to map the existing evidence landscape and identify research gaps, but they were not used as primary sources of data nor included in the final evidence synthesis. In line with scoping review methodology, no formal appraisal of the methodological quality of the included studies was undertaken. Results: Of the 19 articles initially identified, 8 met the inclusion criteria following a rigorous selection process. Key findings indicate that malnutrition on hospital admission is associated with significantly higher rates of 30-day adverse events and in-hospital mortality in patients with TTS. Eating disorders such as anorexia nervosa, by inducing electrolyte disorders and autonomic dysfunction, increase susceptibility to TTS and its complications. The impact of specific deficiencies (e.g., vitamin D) on the deterioration of hemodynamic parameters and the risk associated with renutrition syndrome that may trigger rare variants of TTS has also been documented. Conclusions: Malnutrition, nutritional deficiencies, and eating disorders are significant risk factors and prognosis modifiers in patients with Takotsubo syndrome, leading to an increased incidence of complications and mortality. Early and comprehensive management of nutritional status, including identification and correction of deficiencies and interdisciplinary treatment of eating disorders, is critical to improving clinical outcomes in this vulnerable patient population.

## 1. Introduction

Takotsubo syndrome (TTS), also known as stress-induced cardiomyopathy or “broken heart syndrome,” is an acute and typically reversible impairment of left ventricular systolic function that presents clinically in a manner similar to acute coronary syndrome, despite the absence of significant obstructive coronary artery disease [1].

Takotsubo syndrome accounts for about 2% of all cases of acute coronary syndrome, although in women, this percentage can reach up to 10%, which reflects a clear dominance of women in the patient population—85–90% of patients are women over 60 years of age. In recent years, there has been a systematic increase in the incidence, reaching 7.1 hospitalizations per 100,000 person-years, as well as an increase in the number of diagnoses of atypical variants [2]. The disease is associated with a significant risk of complications, including severe arrhythmias, which occur in about 6% of patients, and a mortality rate comparable to myocardial infarction, especially in the short follow-up period. An increase in the incidence of TTS has also been reported during the COVID-19 pandemic, highlighting the role of emotional and physical stress as key triggers [3,4].

In recent years, there has been a steady increase in TTS diagnoses, especially in postmenopausal women, although this condition can occur in any age group [1,5]. The pathophysiology of TTS remains not fully understood, but a key role is attributed to excessive adrenergic stimulation, dysfunction of coronary microcirculation and disorders of the brain–heart axis [5]. An expanding body of evidence indicates that patients’ nutritional status may have meaningful implications for the clinical trajectory of TTS. In clinical trials, it has been shown that malnutrition occurs in up to half of hospitalized patients with TTS and is associated with twice the risk of 30-day complications, such as acute heart failure, cardiogenic shock or life-threatening arrhythmias [4]. At the same time, there is a growing number of reports describing the co-occurrence of TTS with eating disorders, especially bulimia nervosa and anorexia nervosa, which may predispose to electrolyte disorders, autonomic dysfunction and increased adrenergic reactivity—factors potentially triggering an episode of TTS [1,5].

The importance of nutritional deficiencies and malnutrition in TTS remains poorly understood, even though these disorders can affect myocardial metabolism, stress response, endothelial function, and regenerative processes. In addition, patients with gastrointestinal diseases, absorption disorders or after abdominal surgery may be particularly at risk of developing secondary TTS, which is confirmed by numerous case reports and systematic analyses [2,6]. In light of the increasing number of reports indicating a link between nutritional status and both the risk and clinical course of TTS, there is a need for a comprehensive summary of the available data [7,8]. This scoping review aims to synthetically present the role of malnutrition, nutritional deficiencies and eating disorders in patients with Takotsubo syndrome, with particular emphasis on pathophysiological mechanisms, risk factors, clinical consequences and research gaps requiring further analysis.

## 2. Materials and Methods

### 2.1. Study Design

We conducted a scoping review because our goal was to comprehensively map and synthesize the existing literature on the impact of malnutrition, nutritional deficiencies, and eating disorders in patients with Takotsubo syndrome (TTS). This approach was chosen due to the extensive, complex, and potentially heterogeneous nature of the available literature on the role of these factors in TTS, which may be challenging in the case of a classical systematic review [9].

Our review has been prepared in accordance with the methodology developed by JBI, and is presented in accordance with the PRISMA-ScR guidelines [10,11].

### 2.2. Inclusion and Exclusion Criteria

We formulated a research question that clearly delineated the core PCC elements of the review. This approach enabled the identification of key issues related to the influence of malnutrition, nutrient deficiencies, and eating disorders on TTS, as well as the mapping of reported outcomes and associated risk factors [12].

The inclusion criteria encompassed all published papers, including original research (both observational studies and randomized controlled trials), meta-analyses, systematic reviews, case reports, and narrative reviews, provided they were available in full text, written in English, and conducted in human populations.

The exclusion criteria comprised comments, letters to the editor, book chapters, publications without full-text access, articles published in languages other than English, and studies involving animals.

Population (P): The review included studies describing malnutrition, nutritional deficiencies, or eating disorders in patients diagnosed with Takotsubo syndrome (TTS). Studies focusing on adult patients were mainly considered, although broader reviews covering all age groups were also included if they were relevant to the overarching topic.

Concept (C): The focus was on the impact of malnutrition, nutritional deficiencies (e.g., vitamin D, thiamine deficiency), and eating disorders (e.g., renutrition syndrome, anorexia nervosa) on various aspects associated with TTS. These outcomes included, but are not limited to, incidence of adverse events, in-hospital mortality and complications, as well as mechanisms leading to TTS and prognosis.

Context (C): The studies included in this review were carried out within clinical and research settings focused on the diagnosis, management, and understanding of TTS. This included different healthcare settings, geographic locations, and a variety of approaches to assessing nutritional status. The context also included different types of studies, from controlled trials to systematic reviews and case reports, to provide a comprehensive overview of the evidence.

Study types: The review covered a wide range of study types, including original studies (RCTs, observational studies), systematic reviews, case reports, meta-analyses, and narrative reviews, to provide broad coverage of the existing evidence base.

In the literature, the term Takotsubo syndrome (TTS) is often used interchangeably with the term takotsubo cardiomyopathy (TCM), especially in studies using ICD coding that does not distinguish between the two concepts.

### 2.3. Search Strategy

A comprehensive literature search was performed across the electronic databases PubMed, Scopus, Web of Science, EBSCO (MEDLINE Complete), the Cochrane Library, and Google Scholar. According to the established protocol, the search covered records published between 23 April and 30 April 2026. The search strategy was developed in accordance with PRISMA-ScR and JBI guidelines to ensure methodological transparency and reproducibility [10,11].

The search combined controlled vocabulary (e.g., the terms Medical Subject Headings (MeSH) in PubMed) and free-text keywords related to malnutrition, nutritional deficiencies, eating disorders, and Takotsubo syndrome and their related effects. Filters were applied to restrict the search to human studies, English-language publications, and records available in full text. Eligible study designs included observational research, randomized controlled trials, systematic and narrative reviews, case reports, and meta-analyses. Below is the complete PubMed search strategy, presented as an example of the reproducible search strings applied consistently across all databases.

For Scopus, Web of Science, EBSCO, the Cochrane Library, and Google Scholar, the search strategy was adapted to the specific indexing structure of each database. Boolean operators (AND/OR) and controlled vocabulary, when available, were applied to ensure a comprehensive retrieval of relevant studies. The core search concepts remained consistent across all platforms:

—Concept 1: Nutritional deficiencies/Malnutrition/Eating disorders (e.g., “malnutrition”, “undernutrition”, “nutritional deficiencies”, “eating disorders”, “renutrition syndrome”)
—Concept 2: Takotsubo syndrome (e.g., “Takotsubo cardiomyopathy”, “stress-induced cardiomyopathy”, “broken heart syndrome”, “Tako-tsubo”).

The full list of retrieved records, screening decisions, and reasons for exclusion will be detailed in the PRISMA-ScR flow diagram. Table S1 in the Supplementary Materials summarizes the search strategy.

### 2.4. Data Extraction

To collect key information from the analysed publications, a standardised data extraction form was used, prepared in accordance with the JBI guidelines for scoping reviews and adapted to the specifics of this review [10]. The data extraction process—referred to as “data charting” in the scope reviews—was carried out independently by two reviewers. The PCC (Population, Concept, Context) scheme was used to guide the identification of relevant information in selected studies [9,10].

The form included: bibliographic data (e.g., author, year, country), purpose of the study, characteristics of participants (e.g., number of study participants), type of study (e.g., retrospective observational study, case report, prospective study), and a summary of key results and findings (as shown in Table 1).

Before initiating full data extraction, a pilot test of the extraction form was conducted on a subset of randomly selected publications to ensure that all relevant elements were captured clearly and consistently. Necessary adjustments to the form were made at this stage. Data extraction was then performed by two independent reviewers, who had previously calibrated their approach by simultaneously extracting data from three pilot articles. This calibration aimed to harmonize the interpretation of form fields and reduce the likelihood of discrepancies. Once consistency was achieved, the reviewers proceeded with the full extraction. Any disagreements were resolved through discussion and consensus, with a third reviewer consulted when needed. In accordance with scoping review methodology, no formal inter-reviewer agreement metrics (e.g., Kappa coefficient) were calculated, as the primary objective of this review was to map the existing evidence rather than evaluate the methodological quality of the included studies.

### 2.5. Critical Appraisal Process

A scoping review may summarize the existing evidence without conducting a formal methodological appraisal of the included studies [10]. Thus, a formal critical assessment of the methodological quality of the included studies has not been explicitly conducted, which is consistent with the scoping review methodology that focuses on mapping and summarizing the available evidence.

### 2.6. Process for Including Publications in the Review

**Figure 1:**
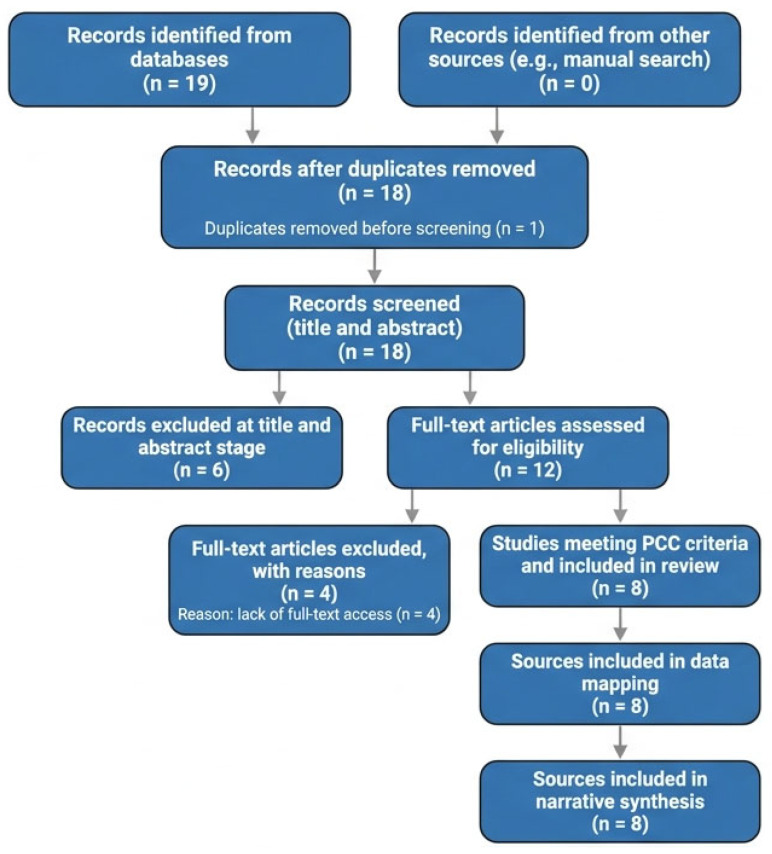
Literature search and selection flowchart for this review.

Our scoping review initially identified a total of 19 articles and ultimately included 8 articles in the final analysis (Figure 1), which examined the roles of malnutrition, nutritional deficiencies, and eating disorders in patients with Takotsubo syndrome. Duplicates (n = 1) were removed, leaving 18 publications. After reviewing titles and abstracts according to the inclusion and exclusion criteria (n = 6), 12 articles remained for full-text screening. Four publications lacked full-text access and were therefore excluded from the review. As a result, after meeting all requirements, 8 publications were included in the review. The studies were performed in various countries, including the USA (n = 3), Japan (n = 3), France (n = 1), and Spain (n = 1), reflecting the wide geographic scope (Table 2).

### 2.7. Selection Process

The selection process was carried out in accordance with PRISMA-ScR guidelines to ensure transparency and reduce the risk of selection bias. All records retrieved from the databases were imported into the Zotero reference management software. Two independent reviewers assessed the articles at each stage. Initially, titles and abstracts were screened independently, followed by full-text evaluation of studies deemed eligible for further review. The selection was performed entirely independently, and any disagreements were resolved through discussion and consensus. When additional input was required, a third reviewer was consulted. The selection process is depicted in a PRISMA-ScR-compliant flow diagram (Figure 1).

The selection process was conducted in accordance with PRISMA-ScR guidelines and comprised four distinct stages:

1. Identification: All records retrieved from the selected databases (PubMed, Scopus, Web of Science, EBSCO, Cochrane Library, Google Scholar) were imported into the bibliography management software.
2. Pre-selection (screening): Two independent reviewers conducted an initial screening of titles and abstracts based on predefined inclusion and exclusion criteria. Publications that clearly failed to meet these criteria were excluded at this stage.
3. Qualification (evaluation of full texts): The full texts of the articles that passed the screening stage were independently assessed by two reviewers. Any disagreements regarding study eligibility were resolved through discussion and consensus, with a third reviewer consulted when necessary.
4. Inclusion: Publications that fulfilled all PCC criteria were ultimately included in the review. The number of records at each stage of the process is presented in a PRISMA-ScR-compliant flow diagram (Figure 1).

### 2.8. Selection of Sources of Evidence Section

Two independent reviewers worked at each stage of the publication qualification process, which guaranteed objectivity and autonomy of the assessment. Any discrepancies were resolved through joint discussion and reaching an agreement, and in disputable situations, the opinion of a third reviewer was used. The entire selection procedure was thoroughly documented in accordance with PRISMA-ScR and JBI guidelines to ensure full transparency and reproducibility [10,11,12].

**Table 1:** Characteristics and key findings of the studies included in this review.

Author, Year	Country	Aim of the Study	Participants	Type of Research	Results and Findings
**Onishi K. et al., 2025** [13]	Japan	investigate the relationship between malnutrition at hospital admission and the occurrence of 30-day adverse events in patients with Takotsubo syndrome (TTS)	124 patients	Retrospective observational study	-Intake malnutrition was associated with an increased rate of 30-day adverse events in patients with TTS -Early identification of malnutrition in clinical practice is important in patients with TTS
**Li P. et al., 2022** [14]	USA	assesses the effect of malnutrition on the in-hospital outcomes of patients with takotsubo cardiomyopathy (TCM)	4733 patients	Retrospective cohort analysis	-Malnourished patients who were admitted with TCM were associated with higher rates of in-hospital mortality and complications compared to those without malnutrition
**Champion S. et al., 2015** [15]	France	evaluation of mechanisms, incidence, treatment and prognosis of TTS	1314 patients (including 20 with TTS)	Retrospective observational study	-One of the mechanisms leading to death in the study group of patients with TTS was malnutrition.
**Dande AS. et al., 2013** [16]	USA	identify the prevalence and clinical consequences of vitamin D insufficiency in patients with takotsubo cardiomyopathy	27 women	Prospective study	-Women diagnosed with takotsubo cardiomyopathy have been shown to have a high incidence of vitamin D deficiency -Lower vitamin D concentrations in patients correlated with worse clinical status, including greater severity of left ventricular dysfunction
**Robles P. et al., 2015** [17]	Spain	Description of the first reported case of inverted Takotsubo cardiomyopathy that was induced by renutrition syndrome	1 woman	Case report	-Renutrition syndrome may cause unusual cardiovascular complications, including supraventricular tachyarrhythmias and Takotsubo cardiomyopathy -The occurrence of cardiac manifestations of re-nutrition syndrome (atrial tachycardia and inverted Takotsubo) may have been influenced by a number of factors, such as hypoglycemia, electrolyte disorders, metabolic disorders and emotional stress—both individually and in combination -Rare variants of Takotsubo cardiomyopathy can be triggered by complex conditions such as renutrition syndrome
**Muto T. et al., 2024** [18]	Japan	presenting and analysing the case of a 67-year-old woman with schizophrenia who developed Takotsubo cardiomyopathy after multiple courses of electroconvulsive (ECT) and ended in death	1 woman	Case report	-Optimization of nutritional support and effective reduction in physiological burden are key elements of management to reduce the risk of death in Takotsubo electroconvulsion-induced cardiomyopathy
**Tagami T. et al., 2016** [19]	USA	Presentation and analysis case of reverse takotsubo cardiomyopathy caused by an eating disorder	1 woman	Case report	-Extreme malnutrition, electrolyte disorders, and chronic metabolic stress can induce reverse Takotsubo cardiomyopathy, confirming that eating disorders are a significant, though rarely reported, trigger of this cardiomyopathy -After the implementation of appropriate nutritional treatment and metabolic stabilization, full normalization of cardiac function occurred, which highlights the crucial role of nutritional interventions in the treatment of eating disorder-induced TTS
**Yanagawa Y. et al., 2013** [20]	Japan	Presentation and analysis case of dementia complicated with Takotsubo cardiomyopathy associated with unconsciousness induced by Wernicke’s encephalopathy	1 woman	Case report	-Psychological stress related to difficult living conditions (loss of electricity, hot and humid apartment, confusion caused by dementia and Wernicke’s encephalopathy) may have been the impetus for the development of Takotsubo’s cardiomyopathy -The administration of thiamine resulted in an improvement in neurological and cardiac status, which emphasizes the need for early diagnosis of vitamin B1 deficiency in similar cases

## 3. Risk Factors and Consequences of Malnutrition, Nutritional Deficiencies and Eating Disorders in Takotsubo Syndrome

In Takotsubo syndrome (TTS), also known as stress cardiomyopathy or “broken heart syndrome,” the interplay between mental, metabolic, and cardiac factors is fundamental to its pathophysiology. In recent years, there has been a growing awareness that patients’ nutritional status and the presence of nutritional deficiencies and eating disorders can significantly affect the risk of TTS, its clinical course and prognosis [6,21]. For this reason, studies classifying a disease entity as TCM have also been included in this review, as long as they have involved the same clinical and pathophysiological populations. In this context, the analysis by Li P. et al. [14] was considered fully consistent with the scope of the review, as it assessed the impact of malnutrition on the clinical course of patients with TTS/TCM, and its results make an important contribution to understanding the role of nutritional status in this disease.

### 3.1. Risk Factors and Pathophysiological Mechanisms

Malnutrition is an alarmingly common phenomenon among patients hospitalized with TTS, the incidence of which exceeds 50% [4,13]. This condition is not only an accompanying phenomenon, but an important prognostic factor, which is associated with twice the risk of 30-day complications, including acute heart failure, cardiogenic shock or life-threatening arrhythmias [4]. Early diagnosis and intervention in the event of malnutrition are therefore crucial for improving the treatment outcomes of patients with TTS [13]. The mechanisms by which malnutrition increases susceptibility to TTS are manifold. Insufficient supply of energy and nutrients leads to cardiac cachexia, weakening of the heart muscle and impairment of its contractile function. Chronic malnutrition also impairs the functions of the immune system, increasing susceptibility to infections and related inflammations, which can be an additional stressor for the body and provoke an episode of TTS, especially in the face of another comorbidity [15,20]. Eating disorders such as anorexia nervosa and bulimia nervosa (bulimia nervosa) are particularly problematic. These disease conditions are often associated with extreme caloric deficiencies and specific nutrient deficiencies, leading to a cascade of negative establishment and electrophysiological effects. Typical for eating disorders are:

a. Electrolyte disorders: Hypokalaemia, hypomagnesaemia, hypophosphataemia, resulting from insufficient supply or loss of electrolytes (e.g., through vomiting, laxative/diuretic abuse). These can lead to significant cardiac conduction disorders and increase the risk of arrhythmias, which are common complications of TTS [5,14].
b. Autonomic nervous system dysfunction: Eating disorders often lead to dysregulation of the sympathetic and parasympathetic systems. Increased activation of the sympathetic nervous system and excessive release of catecholamines, which are a key link in the pathogenesis of TTS, makes the heart more susceptible to emotional and physical stressors [1,5].

At the same time, the role of psychological stress as the main trigger of TTS should be emphasized. Studies have described cases where poorer living conditions, such as inadequate housing conditions or confusion in patients with dementia and Wernicke’s encephalopathy, were an impulse for the development of TTS [20]. In such scenarios, malnutrition and nutritional deficiencies can enhance the biochemical and physiological response to stress, making the body more susceptible to an episode of Takotsubo cardiomyopathy.

### 3.2. Clinical Consequences and Therapeutic Implications

The consequences of malnutrition, nutritional deficiencies, and eating disorders in patients with TTS are serious and go beyond the increased risk of complications. Malnourished patients who develop TTS show higher rates of in-hospital mortality compared to patients without malnutrition [14]. This increased mortality is due to the body’s weakened physiological reserve, impaired ability to heal and regenerate, as well as the aforementioned electrolyte and hemodynamic disorders. For this reason, early identification of malnutrition is becoming a priority in the clinical care of patients with TTS [13]. The process should include a comprehensive assessment of nutritional status, including screening with validated tools (e.g., NRS-2002, MUST), assessment of body composition, biochemical parameters and dietary analysis of intake. The therapeutic implications of these observations are unambiguous:

Correction of nutritional deficiencies: Particular emphasis should be placed on identifying and correcting deficiencies in specific vitamins and minerals, such as vitamin D or thiamine, which may have a direct or indirect impact on cardiac function and stress response [16,20].

According to the scoping review methodology, the mechanisms and consequences presented in Table 2 reflect the observations described in different types of studies, including retrospective analyses and case studies, without attributing to them a causal role. In line with the scope review methodology (JBI, PRISMA-ScR), Table 3 serves as a mapping and synthesizer of the mechanisms and consequences described in different types of studies, including prospective/retrospective analyses and case studies. Observational studies are the main source of clinical data, while case studies are presented only as an illustration of rare or unusual scenarios, without giving them equivalent evidentiary weight.

The results of prospective and retrospective studies are discussed in a separate section as the main source of clinical data. Case studies are presented in a separate subsection, solely to illustrate rare or unusual mechanisms associated with TTS.

**Table 2:** Association between nutritional status and Takotsubo Syndrome: risk factors and clinical implications [13,14,15,16,17,18,19,20].

Category	Risk Factor/Disorder	Pathophysiology/Mechanism of Influence	Clinical Consequences
General malnutrition	Malnutrition on admission to hospital	Cardiac cachexia, weakness of the heart muscle, impairment of systolic function. Impaired immunity, increased susceptibility to infections and inflammation.	Doubled risk of 30-day complications (acute heart failure, cardiogenic shock, arrhythmias). Higher in-hospital and overall mortality. Longer convalescence, worse quality of life. Delayed or incomplete return of left ventricular function.
Eating disorders	Anorexia nervosa, bulimia nervosa	a. Electrolyte disorders: Hypokalaemia, hypomagnesaemia, hypophosphataemia (from supply/loss disorders). b. Autonomic nervous system dysfunction: dysregulation of both the sympathetic and parasympathetic branches, characterized by excessive sympathetic activation and heightened catecholamine release. c. Structural and functional changes of the heart: Atrophy of the heart muscle, reduction in left ventricular mass, reduction in contractility.	Significant cardiac conduction disorders, increased risk of arrhythmias. Increased susceptibility of the heart to emotional and physical stressors. Impaired ability of the heart to respond to sudden stress. Increased susceptibility to fatal disease. High risk group of worse prognosis in TTS.
Specific nutritional deficiencies	Vitamin D deficiency	Role in regulating cardiomyocyte function, inflammatory processes, blood pressure. Weakening of the heart’s ability to adapt under stress.	Worse hemodynamic parameters during a cardiac episode. More unstable clinical course, higher risk of complications.
	Thiamine (vitamin B1) deficiency	It is crucial for energy metabolism. May lead to Wernicke’s encephalopathy and changes in the catecholamine system.	Indirect increase in TTS risk by affecting sympathetic system activation.
	Hypomagnesaemia, hypokalaemia	They lead to serious arrhythmias.	Arrhythmias, especially dangerous in the context of weakened heart muscle during TTS.
Other factors related to nutrition	Renutrition syndrome	Rapid metabolic changes, hypoglycemia, electrolyte disorders, metabolic disorders, emotional distress.	Inducing unusual cardiovascular complications, including Takotsubo cardiomyopathy (e.g., reversed). Supraventricular tachyarrhythmias

## 4. Prognosis for Malnutrition, Nutritional Deficiencies and Eating Disorders in Patients with Takotsubo Syndrome

The clinical prognosis in patients with Takotsubo syndrome (TTS) is complex and depends on many factors, among which nutritional status, the presence of nutritional deficiencies and eating disorders emerge as predictors of significant impact. Although TTS is often seen as a temporary and reversible disorder of left ventricular systolic function, concomitant nutritional problems can significantly alter the natural course of the disease, leading to an increased incidence of complications, longer hospitalization, and even higher mortality. Understanding these relationships is crucial for optimizing therapeutic strategies and improving long-term treatment outcomes [7,13,17].

### 4.1. The Impact of Malnutrition on Short-Term and Long-Term Prognosis

Malnutrition, both in its overt and implicit form, is recognized as an independent prognostic factor in many cardiovascular diseases, and its effect on TTS is no exception. Studies clearly indicate that malnutrition on hospital admission in patients with TTS is associated with significantly higher rates of 30-day adverse events [13]. These include a number of serious complications, such as acute heart failure, cardiogenic shock, severe cardiac arrhythmias (e.g., ventricular fibrillation, ventricular tachycardia), as well as recurrence of TTS episodes [4,18]. The mechanisms underlying the deterioration of the forecast in the short term are multifaceted. Malnutrition leads to weakening of the heart muscle, reducing its functional reserve and ability to cope with the hemodynamic stress accompanying TTS [22]. Malnourished patients often have reduced immunity, which increases the risk of infection and sepsis, which can be an additional stress stimulus and intensify myocardial damage. In addition, protein and energy deficiencies impair repair and regeneration processes, slowing down the return of left ventricular function to normal, which is characteristic of TTS. Li P. et al. showed that malnourished patients admitted with TTS (TCM) were burdened with higher rates of in-hospital mortality and complications compared to patients with optimal nutritional status [14]. This highlights that early nutritional intervention is fundamental to improving survival. In the long term, the impact of malnutrition can also resonate, leading to chronic weakness of the body, increased susceptibility to repeated episodes of TTS, and poorer quality of life [21,23]. Patients who have experienced TTS in a state of malnutrition may require longer recovery and more intensive cardiac rehabilitation. Although Takotsubo’s cardiomyopathy is usually reversible, the accompanying malnutrition can delay or even prevent full recovery of cardiac function, leading to residual left ventricular dysfunction or persistent cardiomyopathy [24].

Specific nutritional deficiencies, often accompanying or occurring independently of general malnutrition, may also have a significant impact on the prognosis in TTS. Vitamin D deficiency, for example, is common among patients with TTS, and, more importantly, is correlated with poorer hemodynamic parameters during a cardiac episode [16,25]. Vitamin D plays a key role in the regulation of the renin–angiotensin–aldosterone system, modulating the inflammatory response, and maintaining the integrity of vascular endothelial function—all of which are involved in the pathophysiology of TTS. Poorer hemodynamic parameters at the onset of the disease, resulting from vitamin D deficiency, may mean a more unstable clinical course, a higher risk of complications and a longer path to full recovery. Systematic vitamin D supplementation in patients with vitamin D deficiency may theoretically improve the prognosis, although this requires further research. Other deficiencies, such as hypomagnesemia or hypokalemia, can lead to serious arrhythmias, which are particularly dangerous in the context of a weakened heart muscle during TTS. Thiamine deficiency, although less frequently described, may affect the energy metabolism of cardiomyocytes and the activation of the sympathetic system, which has a direct impact on the pathogenesis of TTS [20]. Although the direct relationship between thiamine deficiency and prognosis in TTS is not fully established, it is known that this deficiency can lead to Wernicke’s encephalopathy and changes in the catecholamine system, which can exacerbate physiological stress and increase the risk of adverse course.

### 4.2. Prognosis in Patients with Eating Disorders and TTS

Patients with eating disorders, such as anorexia nervosa or bulimia, are a particularly high-risk group when it comes to prognosis in TTS [26,27]. The chronic malnutrition and electrolyte disorders that characterize these conditions make their heart extremely susceptible to catecholamines and stressors. Case report of Tagami T. et al. clearly shows that chronic malnutrition and recurrent aspiration pneumonia weaken the body and may contribute to an increased susceptibility to the fatal course of Takotsubo cardiomyopathy [19]. Similar conclusions can be drawn from the case of Muto T. et al., where adequate nutritional supplementation and reduction in physical stressors were key to avoiding TTS-related death induced by electroconvulsive therapy [18]. The complexity of treatment and the poorer prognosis in this group are also due to the fact that eating disorders are mental problems that require comprehensive and long-term treatment. Cardiac intervention alone, although necessary in the acute phase of TTS, does not eliminate the primary risk factor. Recurrences of eating disorders can lead to recurrences of metabolic, electrolyte, and hemodynamic disorders, increasing the risk of recurrent episodes of TTS. Long-term monitoring, psychological and nutritional support are essential here. The phenomenon of renutrition syndrome, although an acute nutritional complication, also requires special attention in the context of prognosis. Robles P. et al. described a case of Takotsubo-induced reverse cardiomyopathy induced by this syndrome, with accompanying supraventricular tachyarrhythmias [17]. This shows how rapid metabolic changes, triggered by an improperly conducted diet, can lead to acute and life-threatening cardiac complications. This is especially important because renutrition syndrome can occur in malnourished patients at the moment when their condition seems to be improving, suddenly worsening the prognosis [23,27,28].

In light of the above data, an early and comprehensive approach to assessing nutritional status is a key element in optimizing the prognosis in patients with TTS. Screening for malnutrition should be a standard procedure in all patients diagnosed with TTS, and the nutritional interventions implemented should be individualized and tailored to the specific needs of the patient [29]. Strategies to improve the forecast should include:

1. Early identification of malnutrition and eating disorders: Use of validated screening tools and in-depth clinical evaluation [14,22].
2. Correction of nutritional deficiencies: Supplementation of vitamins and minerals, such as vitamin D, under laboratory control [24,25,26,27].
3. Individual nutritional support plan: Introducing nutrition gradually, especially in patients with severe malnutrition, to avoid re-nutrition syndrome [6,29].
4. Interdisciplinary care: Involvement of dieticians, psychologists/psychiatrists (especially in the case of eating disorders), and cardiologists [30].
5. Patient and family education: Increasing awareness of the importance of proper nutrition in the context of regeneration after TTS and prevention of relapse [15,30].
6. Long-term monitoring: In patients with eating disorders, monitoring should be extended to include mental and nutritional aspects to prevent relapses that can put a re-strain on the cardiovascular system [16].

The prognosis in Takotsubo syndrome is significantly modified by the patient’s nutritional status. Malnutrition, specific nutritional deficiencies and concomitant eating disorders are risk factors leading to higher mortality and an increased incidence of complications. Active management of nutritional status, from early screening to comprehensive intervention and long-term support, is critical to improving treatment outcomes and quality of life for patients with TTS. Further prospective research is needed to fully understand complex interactions and develop even more effective prevention and therapeutic strategies [31,32,33,34]. Table 3 presents prognostic impact and modifiers related to nutritional status in takotsubo syndrome. 

**Table 3:** Prognostic impact and modifiers related to nutritional status in takotsubo Syndrome.

Category	Impact on the Forecast (Short and Long Term)	Mechanisms of Forecast Deterioration	Therapeutic Implications/Key Interventions to Optimize Prognosis
General malnutrition	-Significantly elevated rates of 30-day adverse events (acute heart failure, cardiogenic shock, severe arrhythmias, recurrence of TTS) [4,13,18]. -Higher in-hospital mortality [14]. -Longer recovery, more intensive cardiac rehabilitation [21,23]. -Delayed or prevented full recovery of cardiac function, possibility of residual left ventricular dysfunction or persistent cardiomyopathy [24].	-Weakening of the heart muscle and its functional reserve [22]. -Reduced immunity (risk of infection, sepsis) [22]. -Impaired repair and regeneration processes [22].	-Early identification of malnutrition (NRS-2002, MUST) [13,14,29]. -Individualized nutritional support (oral supplementation, enteral/parenteral nutrition) [17,18,19,29]. -Gradual and careful increase in calorie intake to avoid renutrition syndrome [6,17,29].
Specific nutritional deficiencies	-Vitamin D deficiency: Correlation with worse hemodynamic parameters, more unstable clinical course, higher risk of complications [16,25]. -Hypomagnesaemia, hypokalemia: Lead to severe arrhythmias [16,25]. -Thiamine deficiency: May increase physiological stress and increase the risk of adverse course [20].	-Vitamin D: role in regulation of the RAA system, modulation of inflammatory response, endothelial function [16]. -Thiamine: effect on energy metabolism of cardiomyocytes, activation of the sympathetic system [20].	-Identification and correction of deficiencies of specific vitamins and minerals (e.g., vitamin D, thiamine) [16,20,24,25,26,27]. -Systematic vitamin D supplementation (potentially improves prognosis) [25].
Eating disorders (e.g., anorexia nervosa, bulimia nervosa)	-Particularly high-risk group of worse prognosis [26,27]. -Chronic malnutrition and electrolyte disorders increase susceptibility to fatal disease [18,19]. -Risk of recurrence of metabolic, electrolyte and hemodynamic disorders, increasing the risk of recurrent episodes of TTS [26,27].	-Chronic malnutrition and electrolyte disorders make the heart sensitive to catecholamines [26,27]. -Mental disorders requiring comprehensive and long-term treatment [26,27].	-Holistic and interdisciplinary treatment (cardiologist, psychiatrist, psychologist, dietitian) [8,14,30]. -Long-term monitoring and psychological/nutritional support [16,30].
Renutrition syndrome	-Acute and life-threatening cardiac complications (e.g., Takotsubo reverse cardiomyopathy, supraventricular tachyarrhythmias) [17]. -Sudden deterioration of prognosis in patients whose condition appears to be improving [23,27,28].	-Rapid metabolic changes induced by improper nutritional reconstitution [17]. -Hypoglycemia, electrolyte disorders, metabolic disorders, emotional stress [17].	-Gradual introduction of nutrition, especially in patients with severe malnutrition [6,29].
Overall forecast optimization strategy	Improving treatment outcomes and quality of life for TTS patients [31,32,33,34].	Proper assessment and management of nutritional status [29].	-Early identification of malnutrition and eating disorders [14,22]. -Correction of nutritional deficiencies [24,25,26,27]. -Individual nutritional support plan [6,29]. -Interdisciplinary care [30]. -Education of patients and families [15,30]. -Long-term monitoring [16]

## 5. Case Reports Discussed in the Manuscript

The review also identified a set of case studies that, using scoping review methodology, serve as illustrative examples of rare, unusual, or complex mechanisms leading to the development of TTS. While these studies do not constitute a basis for drawing generalized clinical conclusions, they provide important observations regarding specific clinical situations in which nutritional disorders, nutritional deficiencies, or metabolic stresses can trigger an episode of TTS.

The case studies point to several recurring mechanisms: electrolyte and metabolic disturbances as a direct trigger for TTS, extreme malnutrition leading to myocardial weakness and increased adrenergic reactivity, vitamin deficiencies (e.g., thiamine) affecting nervous system function and the stress response, and complex physiological stresses such as refeeding syndrome or repeated ECT sessions that can destabilize the circulatory system. Four case studies, described in Table 2, were included in the analysis, demonstrating various, rare clinical scenarios.

### 5.1. Refeeding Syndrome as a Trigger of Reverse TTS 

Robles P. et al. described the first case of a reverse TTS variant induced by refeeding syndrome. The patient experienced electrolyte disturbances, hypoglycemia, and tachyarrhythmias, which—combined with metabolic stress—led to the development of an atypical form of TTS. The authors emphasized that complex metabolic disturbances can trigger rare variants of stress cardiomyopathy [17]. 

### 5.2. Extreme Malnutrition and Eating Disorders as a Trigger of Reverse TTS 

Tagami T. et al. presented a case of reverse TTS in a patient with severe anorexia. The patient had profound electrolyte disturbances, chronic metabolic stress, and a significant reduction in myocardial mass. After implementing nutritional therapy and metabolic stabilization, full normalization of cardiac function was achieved, highlighting the crucial role of nutritional interventions in this group of patients [19].

### 5.3. Physiological Stress Associated with ECT as a Risk Factor for TTS 

Muto T. et al. described the case of a patient who developed fatal TTS after multiple electroconvulsive therapy sessions. The authors indicated that malnutrition and lack of optimal metabolic support may have increased susceptibility to hemodynamic destabilization during ECT [18]. ].

### 5.4. Thiamine Deficiency and Environmental Stress as Triggering Factors for TTS 

Yanagawa Y. et al. presented the case of a patient with dementia and Wernicke’s encephalopathy, in whom difficult living conditions and vitamin B1 deficiency led to the development of TTS. Thiamine administration improved her neurological and cardiac condition, indicating the importance of early diagnosis of vitamin deficiencies [20]. The case studies presented in this review do not constitute a basis for assessing the prevalence or strength of associations and illustrate rare or complex mechanisms that are not captured in prospective/retrospective studies. Furthermore, these data complement observational data, pointing to potential pathophysiological pathways requiring further investigation. Observational studies (Onishi et al., Li et al., Champion et al., Dande et al.) play a crucial role in interpreting the results of this review, while the case studies serve a descriptive and exploratory purpose, consistent with the purpose of a scoping review.

The case reports presented in Table 4 are illustrative and demonstrate rare or complex mechanisms leading to Takotsubo syndrome. In accordance with scoping review methodology, they do not constitute a basis for assessing the prevalence or strength of associations, but rather complement data from observational studies by pointing to potential pathophysiological pathways requiring further investigation. 

## 6. Limitations and Future Research

This scoping review, although it provides a synthetic picture of the complexity of nutrition issues in TTS, has some limitations resulting from both the nature of the adopted methodology and the limitations of the available literature itself. First of all, the nature of the scoping review, in accordance with the PRISMA-ScR and JBI guidelines, deliberately did not include a formal critical assessment of the methodological quality of the included studies [10,11]. This means that the evidence collected, while a valuable source of information, may vary in terms of the risk of bias and the strength of the conclusions. Many of the included papers are case reports or retrospective observational studies with a small number of participants, which limits the possibility of drawing unambiguous cause-and-effect conclusions and generalizing the results to wider populations. The heterogeneity of the nutritional factors described (from general malnutrition to specific deficiencies and complex eating disorders) and the diversity of methods for assessing them in individual studies posed a challenge in synthesizing the evidence. It is also worth noting that the review focused exclusively on publications in English, which may have led to the omission of relevant studies published in other languages [35].

In light of the above limitations, future research should focus on several key areas. Well-designed prospective cohort studies with a larger number of participants are necessary to accurately determine the prevalence of malnutrition and nutritional deficiencies in patients with TTS and their long-term impact on prognosis. The implementation of standardized and widely accepted screening and diagnostic tools to assess nutritional status in this population will allow for comparison of results between centers and studies [17,29,36]. RCTs are necessary to assess the efficacy and safety of various nutritional interventions (e.g., vitamin D supplementation, nutritional support programs) in improving clinical outcomes in TTS patients. Further research should explore the molecular and cellular mechanisms by which malnutrition and specific nutritional deficiencies affect the pathophysiology of TTS, including the role of the gut-heart axis, inflammatory processes, and mitochondrial dysfunction. In-depth research is needed on therapeutic interventions for patients with TTS and concomitant eating disorders, including interdisciplinary collaboration and long-term monitoring. Given the rapidly evolving knowledge in the field, regular updates of reviews and meta-analyses will be essential to draw on the latest evidence [19,23,35].

The implementation of these research directions will allow for the construction of a more robust evidence base, which will translate into the development of more effective prevention and therapeutic strategies, ultimately improving the care of patients with Takotsubo syndrome and concomitant nutritional problems. The current literature does not allow for drawing far-reaching conclusions of a cause-and-effect nature, and the scope of the review is primarily descriptive. The small number of studies and their heterogeneity point to the urgent need to develop this field, which is one of the key findings of our review.

It is worth noting that a significant limitation of the available literature on the role of malnutrition, nutritional deficiencies and eating disorders in TTS is the fact that a significant part of it is case studies. As our review pointed out, out of 19 publications identified, only 8 met the inclusion criteria, and “the studies were conducted in various countries. including retrospective cohort analyses and case reports”. This means that some of the data is based on single clinical descriptions, which limits the possibility of formulating generalized conclusions about risk factors or prognosis. Therefore, the prognostic conclusions presented in this review are based solely on studies involving larger patient populations, such as the analysis of Onishi et al. (124 patients) or Li et al. (4733 patients), while case studies were only used to illustrate rare pathophysiological mechanisms and atypical variants of TTS. Therefore, further observational studies with greater statistical power are needed to better determine the significance of malnutrition, nutritional deficiencies and eating disorders in the course of TTS.

Case studies are also included in this review, but their role has been deliberately limited to illustrating rare pathophysiological mechanisms and atypical variants of TTS. According to the scoping review methodology, their goal is not to provide predictive or quantitative data, but to map the scope of available evidence. Conclusions about risk factors, prognosis, and clinical consequences were based solely on primary studies involving larger patient populations, such as retrospective analyses by Onishi et al. (124 patients) and Li et al. (4733 patients). For this reason, case reports do not provide a basis for generalized conclusions, but only complement the clinical context in areas where the literature is particularly limited.

## 7. Conclusions

Malnutrition, nutritional deficiencies and eating disorders emerge as important and often underestimated factors that can affect the risk of Takotsubo syndrome (TTS), its clinical course and prognosis. Despite a limited and highly heterogeneous evidence base, available publications consistently indicate that hospital malnutrition is associated with a higher risk of short-term complications and increased in-hospital mortality, while specific deficiencies (e.g., vitamin D or thiamine) and eating disorders may modulate susceptibility to TTS through metabolic, autonomic, and hemodynamic mechanisms. These results highlight the importance of early identification of nutritional disorders and the need to include nutritional assessment in the routine care of patients with TTS.

At the same time, the conclusions of this review should be interpreted with caution. The limited number of studies, their observational nature and the dominance of case reports limit the possibility of formulating causal generalizations. Nevertheless, the synthetic compilation of available data allows for the identification of preliminary, testable hypotheses that can guide future research. Based on the mapped data, future studies should assess whether metabolic stress associated with malnutrition, electrolyte disorders, and impaired myocardial energetics are independent triggers or enhancers of the catecholamine cascade leading to myocardial stupor in TTS.

The available data also suggest that selected micronutrient deficiencies—especially vitamin D and thiamine—may modulate autonomic reactivity and susceptibility of cardiomyocytes to stress, justifying the need for prospective studies targeting these relationships. Cohort studies should also assess whether routine nutritional screening at hospital admission improves short-term and long-term prognosis in patients with TTS, particularly in populations with a high prevalence of malnutrition or eating disorders. In light of the described cases, it is also reasonable to investigate whether rapid changes in glucose–electrolyte balance in the course of renutrition syndrome may constitute a separate pathophysiological mechanism leading to atypical variants of TTS. Axis intersection: psychological stress—autonomic dysregulation—nutritional status provides a promising conceptual framework for future mechanistic research that may help explain the differentiated susceptibility to TTS.

The formulation of these initial hypotheses allows this review to serve as a starting point for more targeted research questions that can be validated in well-designed prospective and intervention studies. Despite the limitations of the current evidence base, the results highlight the clinical relevance of nutritional status in TTS and indicate the need for an interdisciplinary approach combining cardiology, dietetics and psychiatric care.

## Figures and Tables

**Table 4:** Case reports included in the scoping review.

Case Report	Country	Clinical Context	Mechanism/Contributing Factors	Key Clinical Findings	Clinical Implications
Robles P. et al., 2015 [17]	Spain	First reported case of inverted Takotsubo cardiomyopathy triggered by refeeding syndrome	Severe electrolyte disturbances (hypophosphatemia, hypokalemia), hypoglycemia, metabolic stress, emotional stress	Development of supraventricular tachyarrhythmias and inverted TTS during refeeding	Refeeding syndrome may precipitate rare TTS variants; need for careful metabolic monitoring during nutritional rehabilitation
Tagami T. et al., 2016 [19]	USA	Reverse Takotsubo cardiomyopathy induced by anorexia nervosa	Extreme malnutrition, electrolyte depletion, chronic metabolic stress, myocardial atrophy	Reverse TTS with full recovery after nutritional stabilization	Eating disorders represent a significant trigger of TTS; nutritional treatment is essential for cardiac recovery
Muto T. et al., 2024 [25]	Japan	TTS following multiple electroconvulsive therapy (ECT) sessions in a patient with schizophrenia	Physiological burden of repeated ECT, malnutrition, autonomic dysregulation	TTS episode culminating in death	Importance of optimizing nutritional support and reducing physiological stress before ECT
Yanagawa Y. et al., 2013 [20]	Japan	Dementia complicated by Wernicke’s encephalopathy leading to TTS	Severe thiamine deficiency, environmental stress (heat, poor living conditions), confusion	TTS with neurological and cardiac improvement after thiamine administration	Early recognition of vitamin B1 deficiency is crucial; thiamine supplementation may reverse cardiac dysfunction

## Data Availability

No new data were created or analyzed in this study. Data sharing is not applicable to this article.

## Supplementary Materials

The following supporting information can be downloaded at https://www.mdpi.com/article/10.3390/nu18142284/s1. Table S1. Summary of search strategy; Table S2. PRISMA-ScR Checklist.

## Abbreviations

TTS	Takotsubo Syndrome
JBI	Joanna Briggs Institute
PRISMA-ScR	Preferred Reporting Items for Systematic Reviews and Meta-Analyses–Scoping Review extension
PCC	Population–Concept–Context
RCT	Randomized Controlled Trial
MUST	Malnutrition Universal Screening Tool
NRS-2002	Nutritional Risk Screening 2002
TCM	Takotsubo Cardiomyopathy
ECT	Electroconvulsive Therapy
HELLP	Hemolysis, Elevated Liver enzymes, Low Platelets
PET/CT	Positron Emission Tomography/Computed Tomography
SPECT	Single Photon Emission Computed Tomography
ECHO	Echocardiography
FDG	Fluorodeoxyglucose
RAA	Renin–Angiotensin–Aldosterone System
ACS	Acute Coronary Syndrome

## References

[1] Aparisi Á., Uribarri A. (2020). Takotsubo syndrome. Med. Clin..

[2] Kato K., Lyon A.R., Ghadri J.R., Templin C. (2017). Takotsubo syndrome: Aetiology, presentation and treatment. Heart.

[3] Lyon A.R., Bossone E., Schneider B., Sechtem U., Citro R., Underwood S.R., Sheppard M.N., Figtree G.A., Parodi G., Akashi Y.J. (2016). Current state of knowledge on Takotsubo syndrome: A Position Statement from the Taskforce on Takotsubo Syndrome of the Heart Failure Association of the European Society of Cardiology. Eur. J. Heart Fail..

[4] Omerovic E., Redfors B. (2026). Takotsubo syndrome: Pathophysiological insights and innovations in patient care. Nat. Rev. Cardiol..

[5] Cammann V.L., Würdinger M., Ghadri J.R., Templin C. (2021). Takotsubo Syndrome: Uncovering Myths and Misconceptions. Curr. Atheroscler. Rep..

[6] Zeitouni M., Procopi N., Redheuil A., Collet J.P. (2024). Takotsubo syndrome: A cause of reversible microvascular coronary dysfunction. Ann. Cardiol. Angeiol..

[7] Keramida K., Backs J., Bossone E., Citro R., Dawson D., Omerovic E., Parodi G., Schneider B., Ghadri J.R., Van Laake L.W. (2020). Takotsubo syndrome in Heart Failure and World Congress on Acute Heart Failure 2019: Highlights from the experts. ESC Heart Fail..

[8] Madias J.E. (2014). Prevalence of Takotsubo syndrome in men and premenopausal women. Int. J. Cardiol..

[9] Munn Z., Peters M.D.J., Stern C., Tufanaru C., McArthur A., Aromataris E. (2018). Systematic review or scoping review? Guidance for authors when choosing between a systematic or scoping review approach. BMC Med. Res. Methodol..

[10] Peters M., Godfrey C., McInerney P., Baldini Soares C., Khalil H., Parker D. (2015). The Joanna Briggs Institute Reviewers’ Manual 2015: Methodology for JBI Scoping Reviews.

[11] Tricco A.C., Lillie E., Zarin W., O’Brien K.K., Colquhoun H., Levac D., Moher D., Peters M.D.J., Horsley T., Weeks L. (2018). PRISMA extension for scoping reviews (PRISMA-ScR): Checklist and explanation. Ann. Intern. Med..

[12] Peters M.D.J., Godfrey C., McInerney P., Munn Z., Tricco A., Khalil H., Aromataris E., Munn Z. (2020). Chapter 11: Scoping Reviews (2020 version). JBI Manual for Evidence Synthesis.

[13] Onishi K., Matsumura K., Yagi E., Yamada N., Funauchi Y., Kakehi K., Yoshida A., Fujita K., Kawamura T., Matsuzoe H. (2025). Clinical implications of malnutrition on 30-day adverse events in patients with Takotsubo syndrome. J. Cardiol..

[14] Li P., Li C., Mishra A.K., Cai P., Lu X., Sherif A.A., Jin L., Wang B. (2022). Impact of malnutrition on in-hospital outcomes in takotsubo cardiomyopathy. Nutrition.

[15] Champion S., Belcour D., Vandroux D., Drouet D., Gaüzère B.A., Bouchet B., Bossard G., Djouhri S., Jabot J., Champion M. (2015). Stress (Tako-tsubo) cardiomyopathy in critically-ill patients. Eur. Heart J. Acute Cardiovasc. Care.

[16] Dande A.S., Sena S.F., Wasserman H.S., Warshofsky M.K., Belsky J.L. (2013). Prevalence and consequences of vitamin D insufficiency in women with takotsubo cardiomyopathy. J. Clin. Endocrinol. Metab..

[17] Robles P., Monedero I., Rubio A., Botas J. (2015). Reverse or inverted apical ballooning in a case of renutrition syndrome. World J. Cardiol..

[18] Muto T., Kyono H. (2024). A woman with schizophrenia who died due to Takotsubo cardiomyopathy occurring after electroconvulsive therapy. BMC Psychiatry.

[19] Tagami T., Mertens A., Rothschild D., Chowdhury P. (2016). A case of reverse takotsubo cardiomyopathy caused by an eating disorder. J. Cardiol. Cases.

[20] Yanagawa Y., Mikasa M., Nishioka K., Hirano K. (2013). Dementia complicated with Takotsubo cardiomyopathy associated with unconsciousness induced by Wernicke’s encephalopathy. BMJ Case Rep..

[21] Miyoshi T., Higashi H., Amemiya K., Ikeda Y., Yamaguchi O. (2024). Cardiohistological Findings in Renutrition syndrome. Cureus.

[22] Kikuchi K., Yasui-Furukori N., Hasegawa C., Watahiki M., Inoue T., Shimoda K. (2021). Takotsubo cardiomyopathy after hypoglycemia in a patient with anorexia nervosa. Ann. Gen. Psychiatry.

[23] Ederhy S., Dolladille C., Thuny F., Alexandre J., Cohen A. (2019). Takotsubo syndrome in patients with cancer treated with immune checkpoint inhibitors: A new adverse cardiac complication. Eur. J. Heart Fail..

[24] Duval I., Doberentz E., Madea B. (2021). Fatal bleeding after transfemoral coronary angiography in anorexia nervosa. Forensic Sci. Med. Pathol..

[25] Rienas W.M., Pu J., McMahon B., Sarma V., Silverman R., Moscovich T., Borja B. (2025). A Complex Presentation of Anorexia Nervosa and Takotsubo Cardiomyopathy in a Patient of East Asian Descent. Cureus.

[26] Elikowski W., Małek-Elikowska M., Marcinkowski P., Komendzińska-Ognik D., Rzymski S. (2018). Takotsubo cardiomyopathy triggered by profound hypoglycemia in a 39-year-old female with anorexia nervosa: Strain monitoring of left ventricle function recovery. Pol. Merkur. Lek..

[27] Hwang E., Namburar S., Siegel M., Sanchez A. (2024). Takotsubo cardiomyopathy and cardiogenic shock due to hypokalaemic rhabdomyolysis. BMJ Case Rep..

[28] Coen M., Rigamonti F., Roth A., Koessler T. (2017). Chemotherapy-induced Takotsubo cardiomyopathy, a case report and review of the literature. BMC Cancer.

[29] Escudier M., Cautela J., Malissen N., Ancedy Y., Orabona M., Pinto J., Monestier S., Grob J.J., Scemama U., Jacquier A. (2017). Clinical features, management, and outcomes of immune checkpoint inhibitor-related cardiotoxicity. Circulation.

[30] Ederhy S., Cautela J., Ancedy Y., Escudier M., Thuny F., Cohen A. (2018). Takotsubo-like syndrome in cancer patients treated with immune checkpoint inhibitors. JACC Cardiovasc. Imaging.

[31] Kotts W.J., Gamble D.T., Dawson D.K., Connor D. (2022). Psilocybin-induced takotsubo cardiomyopathy. BMJ Case Rep..

[32] Gabarre P., Ruiz P., Chenevier-Gobeaux C., Charpentier E., Soulat-Dufour L., Cohen A., Monnier-Cholley L., Chemali L., François H., Kerneis M. (2022). Inverted Takotsubo Syndrome With HELLP Syndrome: A Case Report. Front. Cardiovasc. Med..

[33] Zmaili M., Alzubi J., Alkhayyat M., Cohen J., Alkharabsheh S., Rana M., Alvarez P.A., Mansoor E., Xu B. (2022). Takotsubo cardiomyopathy in orthotopic liver transplant recipients: A cohort study using multi-center pooled electronic health record data. World J. Hepatol..

[34] Wilson H.M., Cheyne L., Brown P.A.J., Kerr K., Hannah A., Srinivasan J., Duniak N., Horgan G., Dawson D.K. (2018). Characterization of the Myocardial Inflammatory Response in Acute Stress-Induced (Takotsubo) Cardiomyopathy. JACC Basic. Transl. Sci..

[35] Kobylecka M., Budnik M., Kochanowski J., Piatkowski R., Chojnowski M., Fronczewska-Wieniawska K., Mazurek T., Maczewska J., Peller M., Opolski G. (2018). Takotsubo cardiomyopathy: FDG myocardial uptake pattern in fasting patients. Comparison of PET/CT, SPECT, and ECHO results. J. Nucl. Cardiol..

[36] Khalighi K., Farooq M.U., Aung T.T., Oo S. (2015). Takotsubo Cardiomyopathy: A Long Term Follow-up Shows Benefit with Risk Factor Reduction. J. Cardiovasc. Dev. Dis..

