# Supplementary material for: The Role of Malnutrition, Nutritional Deficiencies and Eating Disorders in Patients with Takotsubo Syndrome—Scoping Review
Source: Nutrients. 2026 Jul 12;18(14):2284. doi: 10.3390/nu18142284 (PMC13416205; doi:10.3390/nu18142284)
Supplement: Supplementary file 1 [file nutrients-18-02284-s001.zip › nutrients-4407350-supplementary.pdf]

Table S1. Summary of search strategy

Database	Date of Search	Search String / Controlled Vocabulary	Filters Applied
**PubMed**	23 April 2026	"malnutrition" OR "undernutrition" OR "nutritional deficiencies" OR "vitamin deficiency" OR "eating disorders" OR "refeeding syndrome" AND "Takotsubo cardiomyopathy" OR "stress-induced cardiomyopathy" OR "broken heart syndrome" OR "Tako-tsubo"	English; Humans; Full text; Article types: Clinical Study, Observational Study, RCT, Review, Systematic Review, Meta-analysis, Case reports
**Scopus**	24 April 2026	TITLE-ABS-KEY: "malnutrition" OR "nutritional deficiency" OR "eating disorder" OR "refeeding syndrome" AND TITLE-ABS-KEY ("Takotsubo cardiomyopathy" OR "stress-induced cardiomyopathy" OR "broken heart syndrome"	English; Article; Review; Case reports
**Web of Science**	25 April 2026	"malnutrition" OR "nutritional deficiency" OR "eating disorder" AND "Takotsubo cardiomyopathy" OR "stress-induced cardiomyopathy"	English; Document types: Article, Review; Case reports
**EBSCO (MEDLINE Complete)**	26 April 2026	"malnutrition" OR "nutritional deficiencies" OR "eating disorders" AND "Takotsubo cardiomyopathy" OR "stress-induced cardiomyopathy"	English; Humans; Full text
**Cochrane Library**	30 April 2026	"malnutrition" OR "nutritional deficiency" OR "eating disorder" AND "Takotsubo cardiomyopathy" OR "stress-induced cardiomyopathy"	English; Trials; Reviews

Table S2. PRISMA-ScR Checklist

Section	Item	PRISMA-ScR Requirement	Location in the manuscript
**TITLE**	1	Identify the report as a scoping review	Title page
**ABSTRACT**	2	Structured summary including background, objectives, eligibility criteria, sources of evidence, charting methods, results, and conclusions	Abstract
**INTRODUCTION**	3	Rationale: Describe why the review is needed	Section 1. Introduction
	4	Objectives: Provide an explicit statement of the questions and objectives	Section 1. Introduction (final paragraph)
**METHODS**	5	Protocol and registration (optional): Indicate if a protocol exists	Not registered (stated in Methods)
	6	Eligibility criteria: Specify PCC elements and rationale	Section 2.2. Inclusion and Exclusion Criteria
	7	Information sources: Describe all sources used	Section 2.3. Search Strategy
	8	Search: Provide full search strategy for at least one database	Table S1 + Section 2.3
	9	Selection of sources: Describe the process for selecting evidence	Section 2.6–2.7
	10	Data charting process: Describe methods of data extraction	Section 2.4
	11	Data items: List and define all variables extracted	Section 2.4 + Table 2
	12	Critical appraisal (optional): If conducted, describe methods	Section 2.5 (not conducted)
	13	Synthesis of results: Describe methods for handling and summarizing data	Section 2.4 + Section 3
**RESULTS**	14	Selection of sources: Provide numbers at each stage and reasons for exclusion	Section 2.6–2.7 + Figure 1
	15	Characteristics of sources: Present characteristics of included studies	Section 2.6 + Table 2
	16	Critical appraisal within sources (optional): If conducted, present results	Not applicable (Section 2.5)
	17	Results of individual sources: Present relevant data from each included source	Section 3 + Table 2 + Case reports table
	18	Synthesis of results: Summarize and map evidence	Section 3 + Table 3
**DISCUSSION**	19	Summary of evidence: Summarize main findings and relevance	Section 4. Discussion — Summary of evidence
	20	Limitations: Discuss limitations of the scoping review	Section 4. Discussion — Limitations
	21	Conclusions: Provide overall interpretation and implications	Section 4. Discussion — Conclusions

**FUNDING**	22	Funding: Describe sources of funding for the review	Funding statement (end of manuscript)
